# T-Pattern Detection and Analysis of Football Players’ Tactical and Technical Defensive Behaviour Interactions: Insights for Training and Coaching Team Coordination

**DOI:** 10.3389/fpsyg.2021.798201

**Published:** 2021-12-06

**Authors:** Tiago Fernandes, Oleguer Camerino, Marta Castañer

**Affiliations:** ^1^National Institute of Physical Education of Catalonia, University of Lleida, Lleida, Spain; ^2^Lleida Institute for Biomedical Research (IRBLleida), Lleida, Spain

**Keywords:** performance analysis, interpersonal coordination, defensive phase, tactics, pattern recognition

## Abstract

This article aims to study the coordination of the defenders’ tactical and technical behaviour of successful teams to recover the ball according to contextual variables. A total of 15,369 (480.28 ± 112.37) events and 49 to 12,398 different patterns in 32 games of the 2014 FIFA World Cup’s play-offs were detected and analysed. Results evidenced a T-pattern of the first defender pressuring the ball carrier and his teammates concentrating at the same zone to cover him or space, leading to ball recovery. Field zones, first defender tactical and technical behaviours, and ball carrier first touch constituted opportunities for defenders to coordinate themselves. Moreover, the third defender had a predominant role in his teammates’ temporisation and covering zone behaviours. In the draw, first half, second-tier quality of opponent and play-offs excluding third place and final matches, the ball regularly shifted from upper to lower field zones in short periods, resulting in ball recovery or shot on goal conceded. Defenders performed behaviours farther from the ball carrier, and player-marking were most recurrent to an effective defence. This study’s findings could help coaches give specific tips to players regarding interpersonal coordination in defence and set strategies to make tactical behaviour emerge globally.

## Introduction

Team coordination involves arranging the team members’ behaviours according to their type, timing, and location to achieve the most significant outcome for the team (Eccles, 2010). It is often called interpersonal coordination due to its social nature (Cornejo et al., 2017), which consists of the extend of synchronisation, specification, and stabilisation of behaviours (e.g., body movements or verbal remarks) between two or more people in social interaction (Bernieri and Rosenthal, 1991; Castañer et al., 2010, 2016). For some authors, interpersonal coordination in team sports requires shared cognition to be effective (Gershgoren et al., 2016); for others, it requires shared affordances (Silva et al., 2013; Araújo et al., 2016). The cognitive perspective of coordination function through to the shared cognition or knowledge is helpful to explain discrete tasks. However, it is less valuable to explain activities performed in dynamic and complex environments, focusing on the ecologic and systems perspective (Wharton and Rossi, 2015). Ecologic and systems theory supports the idea that perception-action produces behaviour directly instead of planned movements, and it is integrated into environmental and task constraints (Pinder et al., 2011; Serra-Olivares et al., 2017).

According to previous lines of thought, in sports, the spatial–temporal relationships change continuously, and by consequence, the opportunities to act (Gibson, 1979; Araújo and Davids, 2009; Castañer et al., 2012). The opportunities of actions’ concept, also referred to as affordances, hypothesised that the environment is perceived directly to what is possible for an individual to do (Castañer et al., 2018). For example, a specific opponent’s relative positioning information can be perceived as an opportunity for a player to act (Davids et al., 2005). Thus, team functioning reveals player coordination according to common principles and idiosyncratic behaviours (Araújo et al., 2016). Even so, there is a current discussion that both mechanisms are intimately involved in interpersonal coordination, leading to the development of an integrative perspective to explain interpersonal coordination in situations for which neither shared mental models nor shared affordances can explain clearly (Steiner et al., 2017).

Methodologically, interpersonal coordination can be measured by the following techniques (Cornejo et al., 2017): (i) behavioural coding – tracks global behaviours (ii) video recording; (iii) motion tracking systems – tracks body movements; and (iv) psychophysiological and neurophysiological methods – studies the physiological changes and the neural activity in social interaction. However, video analysis has the advantage to allow the assessment of interpersonal coordination in natural settings (Cornejo et al., 2017). According to the previous authors, the next research of interpersonal coordination should address experimental design sensible to this concept. In addition to the instruments, to study interactive, complex, and dynamic behaviour in sports, researchers have turned to coordinative or collective variables (Hristovski et al., 2014; Low et al., 2020), i.e., “single variables that capture and synthesize the interactive behaviours between the individual parts of a system” (Passos et al., 2014, p. 106).

In football, researchers often use the following collective variables (Clemente et al., 2015; Araújo and Bourbousson, 2016; Sarmento et al., 2018; Low et al., 2020): (i) team centre – lateral and longitudinal cartesian coordinates mean of every player and incorporates variables such as the centroids and “weighted” centroids; (ii) team communication networks – representation and measures of preference and efficacy of players’ connections, and includes variables such as density, centralization, and heterogeneity; (iii) team dispersion – overall spatial distribution of players and includes the variables of stretch index, team spread, surface area, team length per width ratio, and effective space; (iv) team synchrony – consists of the compression dimension of a synergy, i.e., the degree of similarity of behaviours and includes the relative phase and cluster phase measures; and (v) labour division – contribution of players to the team task and integrates the measures of Voronoi, dominant regions, heat maps, major ranges, and player-to-locus distance.

Studies concerning those variables can evidence, for example, that defending types of coupling, in the cases of small-sided games, were consistent, and positioning measures such as surface areas and players’ distances to team centre decreased (Travassos et al., 2014). Teams presented stable patterns concerning defensive, e.g., small stretch index, team width, and effective playing, medium length and team centroid placed in the middle defensive sector and at the right side; and offensive behaviour, e.g., broad stretch index, stable team centre positioned in the central offensive sector at the right and left side, extended length, medium width, and significant players’ dispersion (Ric et al., 2016). Alternatively, general network analysis has shown that high network variables (i.e., total links, density, and clustering coefficient) correlate positively with goals scored. Further, in a multilevel hypernetworks approach, teams revealed changes in dynamics and configurations, e.g., 1v1 and 1v2 interaction of players (i.e., simplices) behind and ahead of the ball position were the most frequent; or that local and global dynamics can be mutually affected (Ribeiro et al., 2020).

Nevertheless, the previous studies, and literature in general, present at least one of the following limitations (Mackenzie and Cushion, 2013; Sarmento et al., 2014; Low et al., 2020): (i) overvaluing of how dyads led to the collective or tactical behaviours, instead of how dyads are formed; (ii) overfocus on attack phase and the spatial–temporal relationships excluding tactical and technical behaviours; (iii) limited analysis of full sided-games or official matches; and (iv) little reflection of complexity and dynamics in data collection and analysis.

In this sense, the defensive theoretical model from Fernandes et al. (2019) that allows the analysis of the defenders’ tactical and technical behaviours (e.g., covering) according to the ball carrier in matches makes possible the study of how dyads are formed. The model also includes situational variables as environmental constraints (e.g., game location or quality of opposition). Weak explanatory power and limited implications have been found when not taking these situational variables into account in the analysis (Mackenzie and Cushion, 2013; Sarmento et al., 2014). Moreover, the T-pattern detection and analysis (TPA) technique facilitates recognising recurrent patterns in information like behaviour events over time, which deals effectively with rare events contrary to the traditional statistical theory (Magnusson, 2017). Recent studies using this technique have shown benefits in studying coordination and sports behaviours (Camerino et al., 2012; Lapresa et al., 2013, 2018; Amatria et al., 2017, 2019; Castañer et al., 2017; Fernández-Hermógenes et al., 2017; Prat et al., 2019; Tarragó et al., 2019).

**FIGURE 1:**
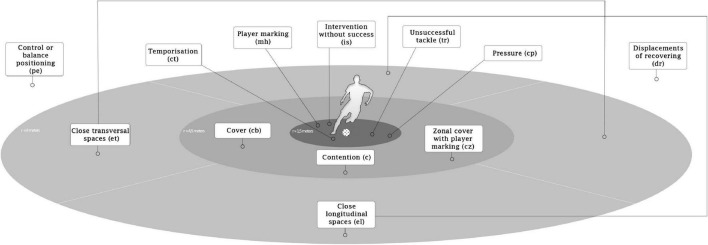
Tactical–technical defensive actions according to distance. The distance and area associated with the tactical and technical behaviours correspond to the ellipse or background’s colour in which they are pointed. However, distance is only one criterion to assess the behaviour. For example, in the “close transversal spaces,” the defender should be positioned to the side and facing the ball carrier, and in the “close longitudinal spaces,” the defender should be placed to the front and facing the ball carrier. For more details, see Fernandes (2017).

Therefore, we hypothesised that particular coordination dynamics of tactical and technical behaviours among defenders are related to team performance (Figure 1). The aims of this study are: (i) to detect and analyse the defensive patterns of tactical and technical behaviours among defenders and (ii) to explore the influences of opponent quality, type and stage competition, match status, and halves in those patterns.

## Materials and Methods

### Participants and Sample

Tactical and technical data from the 2014 FIFA World Cup play-off matches of Germany, Argentina, Netherlands, and Brazil, known as successful teams (Delgado-Bordonau et al., 2013), were collected. Sixteen observations of 12 matches, four observations for each team were analysed. The detailed information about non-ball possessions sample of those matches and per team is available in Fernandes et al. (2020). No sequences were excluded in regular time, and extra-time was not included, procedures similar to previous works (Barreira et al., 2015).

### Instrument and Variables

The Soccer-Defence (SOC-DEF) Theoretical Dynamic System Model was used to collect the data. The criteria and categories’ definitions, validity, and reliability are presented elsewhere (Fernandes et al., 2019). We used the Lince 1.4 recording software (Gabin et al., 2012) and LINCE PLUS (Soto et al., 2019, 2021) to implement the instrument, record the behaviours and organise the data. FIFA database provided tactical and broadcast cameras, which were utilised to decrease data exclusion and ambiguous actions.

### Design and Procedures

This study consists of a nomothetic (i.e., plural unit), followed-up (i.e., diachronic), and multidimensional (i.e., multiple responses) design, which corresponds to the fourth quadrant of the observational methodology (Chacón-Moscoso et al., 2018). The sample is made of sequences of the non-possession of the ball from the play-offs matches of semi-finalists teams, according to the definitions of Barreira et al. (2012). Multi-codes of behaviours were recorded at the first touch, the following three touches, and the last touch (Fernandes et al., 2019).

The data were recorded by a football performance analyst with 8 years of previous experience coded the data of all matches. The operator followed the procedures of the instrument protocol severely to ensure measurement blinding.

### Data Analysis

According to previous research (Lapresa et al., 2013, 2018; Amatria et al., 2017), the following search parameters were set: free critical interval, significance level at 0.005, minimum occurrence equal or more than three events, lumping factor of 0.90, types of randomisations (shuffling and rotation), and 2000 randomisations (1/0.005 × 10). An empirical selection of the criteria was made to decrease the event types and increase the number of events, i.e., reduce variability. Pattern recognition analysis on each coded interaction session and criteria selection was performed using THEME Edu v6. THEME is a software package featuring algorithms that process the enormous range of combinatorial patterns underlying behaviours; it compares all behavioural patterns and retains only the most complete ones.

## Results

**FIGURE 2:**
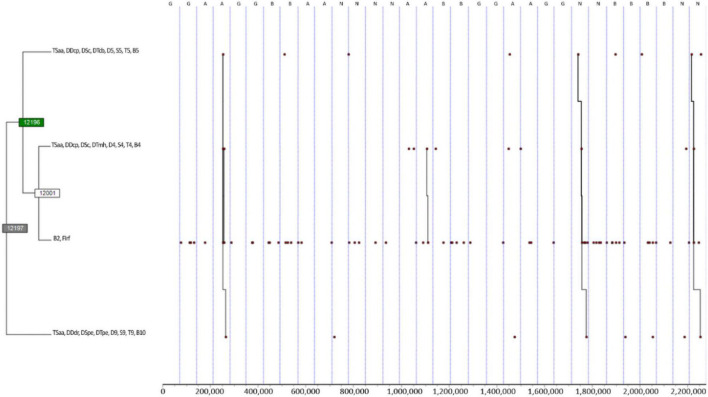
T-pattern detection and analysis of the first, second, and third defender’s tactical–technical actions and field zones, type of subphase, ball field zone, and end of defensive phase, using a lumping factor of 0.90 and minimum occurrence of 3. Pattern: ((TSaa, DDcp, DSc, DTcb, D5, S5, T5, B5 (TSaa, DDcp, DSc, DTmh, D4, S4, T4, B4 B2, FIrf)) TSaa, DDdr, DSpe, DTpe, D9, S9, T9, B10); A, Argentina; B, Brazil; B2, ball in the central strip and ultra-defensive sector field zone; B4, ball in the left strip, defensive sector, and defensive midfield field zone; B5, ball in the central strip and defensive sector field zone; B10, ball in the central strip and offensive sector field zone; D4, first defender in the left strip, defensive sector, and defensive midfield field zone; D5, first defender in the central strip and defensive sector field zone; D9, first defender in the central strip and offensive midfield sector field zone; DDcp, first defender pressure; DDdr, first defender displacements of recovering; DSc, second defender contention; DSpe, second defender control or balance positioning; DTcb, third defender cover; DTmh, third defender player marking; DTpe, third defender control or balance positioning; FIrf, end of defensive phase by shot off goal conceded; G, Germany; N, Netherlands; S4, second defender in the left strip, defensive sector, and defensive midfield field zone; S5, second defender in the central strip and defensive sector field zone; S9, second defender in the central strip and offensive midfield sector field zone; T4, third defender in the left strip, defensive sector, and defensive midfield field zone; T5, third defender in the central strip and defensive sector field zone; T9, third defender in the central strip and offensive midfield sector field zone; TSaa, delay subphase.

**FIGURE 3:**
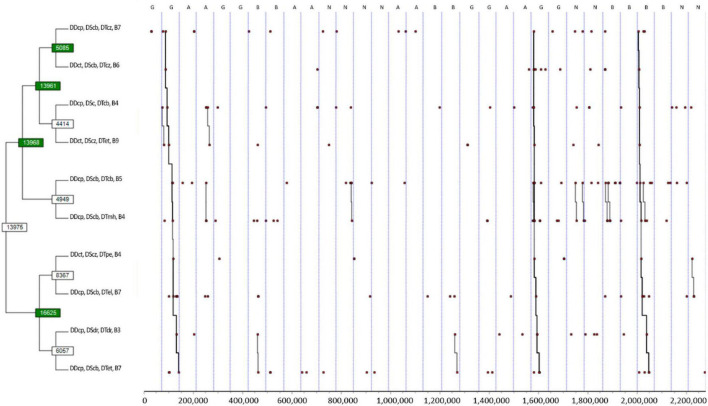
T-pattern detection and analysis of the first, second, and third defender’s tactical–technical actions and ball field zones, using a lumping factor of 0.90 and minimum occurrence of 3. Pattern: ((((DDcp, DScb, DTcz, B7 DDct, DScb, DTcz, B6) (DDcp, DSc, DTcb, B4 DDct, DScz, DTet, B9)) (DDcp, DScb, DTcb, B5 DDcp, DScb, DTmh, B4)) ((DDct, DScz, DTpe, B4 DDcp, DScb, DTel, B7) (DDcp DSdr, DTdr, B3 DDcp DScb, DTet, B7))); A, Argentina; B, Brazil; B3, ball in right strip and ultra-defensive sector field zones; B4, ball in the left strip, defensive sector and defensive midfield field zone; B5, ball in the central strip and defensive sector field zone; B6, ball in the central strip and defensive midfield field zone; B7, ball in the right strip, defensive sector, and defensive midfield field zone; B9, ball in the central strip and offensive midfield sector field zone; DDcp, first defender pressure; DDct, first defender temporisation; DScb, second defender cover; DScz, second defender zonal cover with player marking; DSdr, second defender displacements of recovering; DTcb, third defender cover; DTcz, third defender zonal cover with player marking; DTdr, third defender displacements of recovering; DTel, third defender close longitudinal spaces; DTet, third defender close transversal spaces; DTmh, third defender player marking; DTpe, third defender control or balance positioning; G, Germany; N, Netherlands.

**FIGURE 4:**
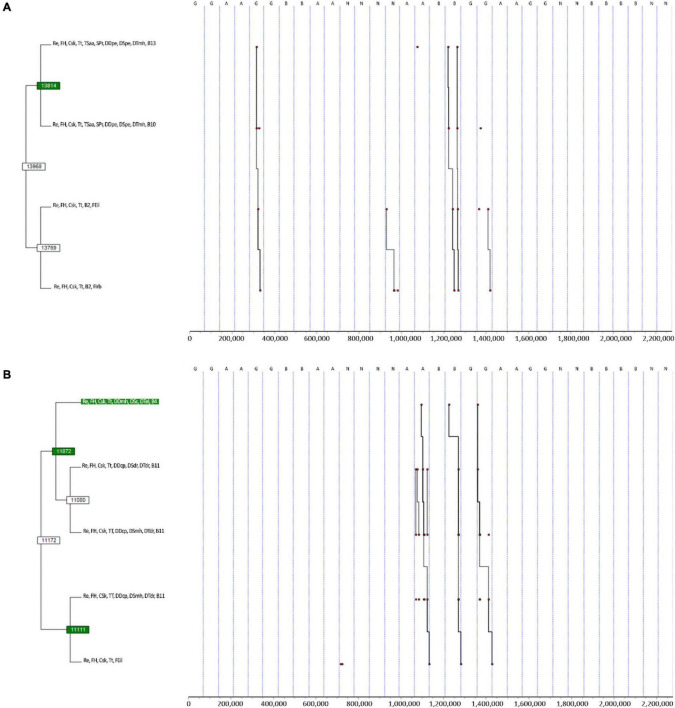
T-pattern detection and analysis of the first, second, and third defender’s tactical–technical actions and ball field zones, with and without the type of subphase and centre of the game, according to match status, opponent quality, type and stage of competition, and halves, using a lumping factor of 0.90 and minimum occurrence of 3. Pattern **(A)** ((Re, FH, Csk, Tt, Tsaa, SPr, DDpe, DSpe, DTmh, B13 Re, FH, Csk, Tt, TSaa, SPr, DDpe, DSpe, DTmh, B10) (Re, FH, Csk, Tt, B2, FEil Re FH, Csk, Tt, B2, FIrb)); Pattern **(B)** ((Re, FH, Csk, Tt, DDmh, DSc, DTel, B4 (Re, FH, Csk, Tt, DDcp, DSdr, DTdr, B11 Re, FH, Csk, Tt, DDcp, DSmh, DTdr, B11)) (Re, FH, Csk, Tt, DDcp, DImh, DTdr, B11 Re, FH, Csk, Tt, FEil)); A, Argentina; B, Brazil; B2, ball in the central strip and ultra-defensive sector field zone; B4, ball in the left strip, defensive sector, and defensive midfield field zone; B10, ball in the central strip and offensive sector field zone; B11, ball in the right strip, offensive midfield, and sector offensive sector field zone; B13, ball in the central strip and ultra-offensive sector field zone; Csk, play-offs excluding third place and final; DDcp, first defender pressure; DDmh, first defender player marking; DDpe, first defender control or balance positioning; DSc, second defender contention; DSdr, second defender displacements of recovering; DSmh, second defender player marking; DSpe, second defender control or balance positioning; DTdr, third defender displacements of recovering; DTel, third defender close longitudinal spaces; DTmh, third defender player marking; FEil, end of defensive phase by interruptions and laws infractions; FH, first half; FIrb, end of defensive phase by shot on goal conceded; G, Germany; N, Netherlands; Re, draw; SPr, relative numerical inferiority; TSaa, delay subphase; Tt, tier 2.

The total number of event types, i.e., the various combinations of players’ behaviours, analysed in the 32 games was 15,073 (478.00 ± 112.10) from a total duration (i.e., unit of analysis) of 2,277,370 (71,167.81 ± 2448.59). Only 296 event types were repeated; however, this value increased with the criteria selection ranging between 3432 and 11,305. THEME software detected a minimum of 49 and a maximum of 12,398 different patterns in the five sessions performed. The results of the T-patterns of the five sessions are presented in Figures 2–4.

Figure 2 shows that in the Delay (TSaa) subphase, when the first defender pressured the ball carrier (i.e., DDcp) at the centre of the entry of the box (i.e., zone five), the second defender did contention (i.e., DSc), and the third defender covered (i.e., DTcb), this was a precedent of the ball going to the left strip of the medium defensive sector (zone four) with the first, second and third defenders doing pressure (DDcp), contention (DSc) and player marking (DTmh), respectively. However, if a ball went inside the box (i.e., B2), it could result in a shot off goal conceded (FIrf). On the other hand, if the defenders performed efficiently, the opponent team could retreat to their midfield (zone 9 and 10), which triggered the defending team to perform recovering and positioning displacements (i.e., DDdr, DSpe, and DTpe). The T-pattern was most seen in both the Netherlands and Argentina.

The analysis without the defenders’ location resulted in a more informative T-pattern, as shown in Figure 3. The T-pattern starts with the first defender pressuring the opponent with the ball (i.e., DDcp), the second and third defenders covering (i.e., DScb) the first defender and the zone (i.e., DTcz) being the right strip of the field (i.e., B7). Then, the first defender temporised the opponent action (DDct) when the ball went to the central zones (i.e., B6) and returned to pressure in the sides of the field (i.e., B4) with the second and third defenders performing contention (i.e., DSc) and covering (i.e., DTcb). If a ball moved to the central zones of the opponent field (i.e., B9), the first defender restrained himself again (i.e., DDct) and the second defender and third defender adopted behaviours with emphasis to space (i.e., zonal covering and closing transversal spaces). Shifting the ball to zones four and five made the first defender pressure and the second defender covered the first defender’s pressure (i.e., DDcp and DScb). In these dynamics, the third defender performed cover (DTcb) and player marking (DTmh). Next, if the ball continued in the same zone (i.e., B4), the first defender temporised the ball carrier actions (i.e., DDct), and the second and third defenders focused on the space (i.e., DScz and DTpe). When this occurred, shifts to the other side of the field (i.e., B7 and B3) were frequent, and the first defender was making pressure (DDcp), the second and third defenders doing covering (DScb) and closing longitudinal spaces (DTel). However, getting into zone 3, their behaviours changed to the displacement of recovering (i.e., DSdr and DTdr). More or fewer parts of the pattern were detected in all teams, but the complete ones are evidenced in Germany and Brazil.

Further, the analysis considering the contextual variables (Figure 4) detected T-patterns in the draw (Re), first half (FH), second-tier quality of opponent (Tt), and play-offs excluding third place and final (Csk). In Figure 4A, when the subphase and the numerical relationship (i.e., centre of the game) were considered, we can see that teams performed more actions far from the ball carrier (i.e., DDpe and DSpe), which could result in the opponent moving the ball along the zones near to their goal (i.e., B10 and B13) and consequently lose it for infraction of the laws (FEil) in the other side of the field (i.e., B2). On the other hand, it could also result in a shot on goal (FIrb). Then, when the subphase is not considered (Figure 4B), at the same situation, i.e., Re, FH, Tt, and Csk, but in zone 4, the successful teams’ first defender performed player marking (DDmh), while the second and third defenders performed contention (DSc) and closed longitudinal spaces (DTel). The ball going to zone 11 encouraged the first defender to perform pressure (DDcp), and both the second and third defenders made displacements of recovering (DSdr and DTdr). Sequentially, the second defender performed player marking (DSmh), and the third defender continued to recover the positioning (i.e., DTdr). Despite this moment of instability, the result was recovering the ball by opponents’ infraction of game laws (i.e., Feil). Brazil, Argentina, and Germany demonstrated this same dynamic pattern.

## Discussion

All aims proposed for this article were accomplished as the defensive tactical and technical behaviours patterns among players were identified and discriminated, and the influences of opponent quality, type and stage competition, match status, and halves on those defensive patterns were explored.

The number of events shows how heterogeneous is the game of football. Spatiotemporal data do not reveal direct or clear information on the tactical and technical behaviour of the player. In other words, the same coordinates can have completely different meanings since the player can perform different actions in the same space.

The T-patterns of Figure 2 had the most event and event types, meaning that great complexity was considered. Players placed on the sides, especially in the left side of Argentina and Netherlands, coordinate in the first touch of the ball carrier; this could be due to the fact that the delay subphase is the most common in football matches (Fernandes et al., 2020); in other words, teams often play in one and two touches. Not having patterns considering more than one and two touches indicates the players’ significant behaviour variability indirectly, i.e., loss of coordination, but deductive reasoning should be tested empirically.

Also, it seems that the coordination of the players following the reasoning of pressuring the ball carrier and having teammates saving their back seems coherent with the concentration of players at the same zone (i.e., pressure zones) and indicates the successful end of the defensive phase even if it is an indirect type of ball recovery. Despite the methodological differences, one can make a parallelism with the results found in European professional football, specifically defensive effectiveness against shooting conceded is correlated with winning matches (Brito Souza et al., 2019), and defensive pressure decreases the probabilities of scoring a goal (Gonzalez-Rodenas et al., 2020). Moreover, the significant frequency and complexity in the results found in Figure 3 resulted from the criteria selection, which means that partial information of the game was lost. However, the results generally expressed how defenders coordinate themselves related to ball location, mainly in the right and left sides of the defensive and midfield sectors. Interestingly, previous research using T-patterns in the attack phase, but in a single elite club, showed opposed patterns, making sense considering the different phases (Camerino et al., 2012). However, one should be aware of the methodological differences and limitations of this comparison. Either way, zones in this study showed affordances for the coordination of the players, at the right strip and ultra-defensive sector seems to be a zone of 1v1 situation that the first defender pressured even without the backing of the second and third defenders. Despite the methodological differences, one can compare to the results of the studies of Laakso et al. (2017) and Laakso et al. (2019), who found lower values of interpersonal distance in the middle and left zones in 2v1 and 1v1 (isolated) situations, respectively.

However, the typical behaviour of the first and second defenders at the right and the left strip was to pressure from the defensive sector and defensive midfield zones, but only when the third defender was near to them. It still, it depends on the third defender – if the third defender was far from the centre of the game, the defenders adopted temporisation and covering zone behaviours. Also, it seems that the third defender was responsible for conditioning the next move of the attack. For instance, while in his team’s midfield, he appeared to be concerned about avoiding the ball from getting between the lines, in the opposite midfield, he was focused on channelling the attack to one side of the field, reducing the attacking team’s options to proceed.

Nevertheless, the tactical and technical behaviours differed from the right and left sides. The difference of the second defender’s behaviour between the right and left strip can be explained by the difference between the roles and characteristics of the right and left players (Laakso et al., 2017, 2019) or different tactics set by the coach to the wider players, giving emphasis to what Gesbert and Hauw (2019) addressed about the importance of phenomenological data in studying team coordination. On the particular zone 9, the third defender seems to be preoccupied with not letting the ball go to a side of the field, which could mean that the defending team was trying to manipulate the attacking team to go to a specific zone of the field or trying to create a zone of pressure.

The results of the analysis of the contextual variables seem to be related to the size of the sample, as the tier 2 of opponent quality of opponent (i.e., TT), draw (i.e., Re), and play-offs excluding third place and final (i.e., Csk) were the variables where most non-possessions of the ball were found. On the other hand, the halves variable results do not support this argument entirely, as they are very similar. Still, the more significant time in defence in Csk defense to group stage result corroborates the one found by Alves et al. (2019) in the 2018 FIFA World Cup. On the non-possessions of this study, the shift of zones from upper field to lower zones and the defenders’ distance to the ball indicates that the attack proceeded with a long pass, resulting in either an effective or ineffective end of the defensive phase. The information of this study does not support a reasoned explanation for this result. Thus, more research is needed to understand which mechanisms relate to long passes’ defence effectiveness.

Further, it seems that the first defender is player marking the ball carrier, the second defender is making contention, and the third defender is closing the longitudinal space, i.e., space which penetration or through ball can happen (Zani et al., 2021), against teams who lost in the eight-or quarterfinals in the first half, induce the ball going to the opposing side of the field. In there, the pressure and player marking from the first and second defenders emerge as an effective end of the defensive phase. These results align with the findings of Frias and Duarte (2014), who found different trends of spatial–temporal variables according to man-to-man (or player marking) and zone marking. However, their study results suggest that zonal defence is most effective, which opposes the results of this study, as one can argue that winner teams are supposed to have a more effective defence, or can player marking be enough to win against lower teams?

An explanation for player marking against lower teams is that fewer resources are required to visual search in zonal defence and thereby it is less mental demanding (Fan et al., 2015; Krzepota et al., 2016). Also, it can be effective against less skilful and less tactically intelligent players, as previous studies showed, despite the methodological differences, that skilled players produced the most unpredictable behaviour. Besides, at the sides of the field, the attacking players have fewer degrees of freedom (i.e., the lateral lines limit their space). On the other hand, and quite paradoxically, player marking can be immensely challenging physically (Casamichana et al., 2015) and thus makes sense that this happens more often at the first half of the match as football players tend to have higher values in physical and physiological variables (Ngo et al., 2012; Torreño et al., 2016).

Future research should extend this observation for all 11 players and combine them with spatiotemporal data and social network theory (Ribeiro et al., 2020). However, there are many behaviours that an individual player can perform in football, but there are also too many combinations of behaviours among players. Also, it seems relevant for practice weighting how well an action is performed (Pizarro et al., 2020). Besides, most researchers choose not to incorporate all the complexity because it is likely that the results would not have a coherent interpretation or interpretation would not be possible. This research has the limitation of a small sample compared to the complexity included. In research often the Occam’s razor argument is raised (Riesch, 2010), meaning, practically in football, for example, that researchers should include the minimum behaviours that describe reality, i.e., the behaviour that makes a difference and not only the regular ones. Thus, we argue that the decision to eliminate variables and have less data is an advantage rather than a limitation; plus, it could be compared to the techniques of selection of variables in traditional statistics (Heinze et al., 2018). Thus, expert knowledge is required to understand the data and its implications. This complexity makes the computation and statistical analysis extremely hard; however, researchers should consider the oversimplification bias (Arp et al., 2018).

Similar data to the one of this study can be challenging to collect and, nowadays, there are techniques to collect data (e.g., automatic tracking systems) quicker and exhaustively. However, those instruments still do not evidence the number and the quality of players’ actions completely. Therefore, further studies should consider incorporating more player behaviours in automatic systems and big data procedures (Goes et al., 2020). It can also be argued that the FIFA World Cup 2014 data are outdated and that rules have changed since (Augste and Cordes, 2016). However, we argue that previous editions of tournaments and specifically successful teams can provide relevant and tactical knowledge as it underlines into regular principles of the game’s essence (e.g., Delgado-Bordonau et al., 2013). For instance, some tactical principles of the Netherlands of the 1970s still influence today’s coaches and players (Winner, 2012).

Finally, this study’s findings could help coaches give specific tips to players to have interpersonal coordination in defence. Further, such information can be delivered either to elite players or young players. For instance, the patterns found can be followed as defensive principles or heuristic strategies but in an integrative approach of cognitive and ecologic perspectives (Steiner et al., 2017). In the same line of thought, the demands of how dyads are formed among defenders can help coaches anticipate settings and set strategies to promote favourable tactical team behaviour.

## Data Availability Statement

The raw data supporting the conclusions of this article will be made available by the authors, without undue reservation.

## Author Contributions

TF: introduction, data collection, and discussion. OC and MC: methods and data analysis. All authors contributed to the article and approved the submitted version.

## Conflict of Interest

The authors declare that the research was conducted in the absence of any commercial or financial relationships that could be construed as a potential conflict of interest.

## Publisher’s Note

All claims expressed in this article are solely those of the authors and do not necessarily represent those of their affiliated organizations, or those of the publisher, the editors and the reviewers. Any product that may be evaluated in this article, or claim that may be made by its manufacturer, is not guaranteed or endorsed by the publisher.
